# Quercetin improves lacrimal gland function through its anti-oxidant actions: Evidence from animal studies, and a pilot study in healthy human volunteers

**DOI:** 10.3389/fnut.2022.974530

**Published:** 2022-10-06

**Authors:** Takaaki Inaba, Mayumi Ohnishi-Kameyama, Ying Liu, Yasuhisa Tanaka, Masuko Kobori, Shusaku Tamaki, Tomotaka Ito, Kazunari Higa, Jun Shimazaki, Kazuo Tsubota

**Affiliations:** ^1^Department of Ophthalmology, Keio University School of Medicine, Tokyo, Japan; ^2^Department of Ophthalmology/Cornea Center, Tokyo Dental College Ichikawa General Hospital, Ichikawa, Japan; ^3^Food Research Institute, National Agriculture and Food Research Organization (NARO), Tsukuba, Japan; ^4^Tsubota Laboratory, Inc., Tokyo, Japan

**Keywords:** quercetin, dry eye, tear secretion, lacrimal gland, anti-oxidant

## Abstract

Anti-oxidant properties of polyphenols have been gaining medical attention as a preventive factor against aging and/or lifestyle diseases. In this study, we examined the anti-oxidant activity of quercetin improved tear function through its effects on the lacrimal gland in mice and humans. Six week-old diabetic mice, a model for decreased tear production, were fed for 12 weeks *ad libitum* with an experimental diet containing 0.5% quercetin. As a result, the tear volume was significantly improved compared to the control, despite no changes in body weight, food intake, lacrimal gland morphology or biochemical serum parameters. Moreover, significantly higher SOD-1 and SOD-2 protein levels were detected in the lacrimal glands of quercetin-treated mice by western blot. In addition, quercetin treatment of mouse corneal cell lines exposed to oxidative stress resulted in dose-dependent inhibition of ROS production and enhanced cell survival. Finally, we examined quercetin pharmacokinetics, specifically its presence in serum and tears subsequent to onion consumption in healthy volunteers, and found that the distribution of quercetin and its metabolite shifted from serum to tear following onion intake. An improvement in tear film stability also resulted following the intake by these healthy volunteers of a new, quercetin-rich onion cultivar (”Quergold”) in powder form. These results suggested that quercetin improved tear function through its effects on the lacrimal gland in mice and humans.

## Introduction

The structure and function of the eye is readily affected by exposure to ultraviolet radiation and lighting. Ocular tissues including cornea, lens, and retina are consistently under considerable oxidative stress caused by regular exposure to light. This prospect for reduced visual function from light-induced damage is considered a serious problem because it severely threatens quality of life. Further, life style factors such as the recent information technology-generated increase in Visual Display Terminal (VDT) work, and the prevalence of metabolic syndrome resulting from excess caloric intake exacerbate damage to ocular tissues (1, 2). Indeed, there are many reports of reduced tear secretion in patients with type-2 diabetes, and we have confirmed this reduction in diabetic mouse models (3). In response, expectations for functional food factors (nutraceuticals) to alleviate oxidative stress have been rising, and early proactive dietary intervention including use of dietary supplements has been gaining medical attention as a potential treatment modality for age-related dry eye conditions (4–7).

Quercetin has attracted notice for its remarkable antioxidative action in preventing metabolic syndrome (8). Quercetin possesses one of the most potent antioxidant activities in the flavonoid class of molecules, identifying it as a functional food factor (9). For instance, 1-week consumption of an olive oil extract of onion has been reported to significantly decrease systolic blood pressure (10), and also to improve red blood cell and platelet aggregation, in patients with mild hypertension (11). In clinical reports, the antioxidative activity of quercetin for its potential in preventing and/or alleviating various eye diseases for which aging and oxidative stress are the key risk factors (12–14). The superoxide dismutase (SOD) enzymes comprise a major antioxidant system, and deficiency of Cu, Zn-superoxide dismutase-1 (SOD-1) in mice leads to dry eye disease that resembles accelerated aging (15). These studies, as well as, many *in vitro* studies, suggest the potential benefits of quercetin, as a representative polyphenol, in clinical application for prevention and treatment of dry eye disease (16).

In this study, we investigated the relationship between oxidative stress reduction by quercetin and tear secretion, as well as the potential of quercetin, a polyphenol antioxidant, as an effective food factor for intervention against dry eye diseases. In a pilot study to further clarify the effectiveness of quercetin against dry eye, we also tested the pharmacokinetic of quercetin compartmentalization into tears following oral ingestion, to determine the effective concentration.

## Materials and methods

### Mouse study

All the animal experiments were conducted in accordance with the Association for Research in Vision and Ophthalmology (ARVO) Statement for the use of animals in research. All experiments were approved by the Japanese Physiological Society’s guidelines for animal care and the animal experimental committees at KEIO University (approval number: 08067). Mice were maintained on a 12-h light–dark cycle, with the dark cycle occurring from 8:00 P.M. to 8:00 A.M in a specific-pathogen-free environment. Six-week-old female db/m (BKS.Cg-m + / + Lepr^db^/Jcl) and db/db (BKS.Cg- + Lepr^db^/ + Lepr^db^/Jcl) mice were obtained from CLEA Japan (Tokyo, Japan) (17).

The db/db mice (*n* = 20) were randomly divided into 2 groups. The db/m (*n* = 10) and db/db group (*n* = 10) were fed *ad libitum* with Rodent Diet CE-2 (CLEA, Tokyo, Japan) for 20 weeks. The quercetin group (*n* = 10) was fed with the experimental diet formulated by adding 0.5% (w/w) quercetin (Cayman, Michigan, USA) to the CE-2 from 6 weeks of age. All the mice underwent tear collections for the examinations and were sacrificed at 20 weeks of age. Body weight and total food intake were recorded weekly, and the daily food intake was calculated. Lacrimal gland and whole eyes were collected for histopathology analysis and western blotting. Lacrimal gland weights were measured after sacrifice, at which time serum samples were collected for biochemical analysis (Oriental Yeast Co., Ltd., Tokyo, Japan).

### Measurement of tear secretion volume

Basal tear secretion volume was measured with a cotton thread test by using a phenol red-impregnated thread (Zone-Quick, Showa Yakuhin Kako, Tokyo, Japan) (18). The mice were restrained without anesthesia, and a cotton thread was placed on the temporal side of the lower eyelid margin for 30 s. The length of the moistened fraction of the thread was measured. Measurements were obtained for both the right and the left eyes. Tear volume was adjusted for the weight of each lacrimal gland to obtain the tear secretion ratio.

### Histopathology

For histopathology analysis, following dissection from the eye, the lacrimal glands, cornea, conjunctiva and meibomian gland were fixed as part of the eyelid in 10% formalin neutral buffer solution (Wako, Osaka, Japan) overnight at 4°C. The tissues were rinsed and dehydrated using conventional methods, and embedded in paraffin. Five-μm microtome sections were obtained and stained with hematoxylin and eosin (H/E).

### Western blotting

Individual lacrimal glands were homogenized in Pro-prep (iNtRON Biotechnology, Kyungki-Do, South Korea), using mechanical dispersion (T-25; IKA, Stauffen, Germany). The protein concentrations of the lacrimal gland homogenates were measured using the BCA kit (Thermo Fisher Scientific, MA, USA) according to the manufacturer’s instructions. Lacrimal gland lysates were combined with sample buffer [125 mM Tris–HCl, pH 6.8; 20% glycerol; 4.0% sodium dodecyl sulfate (SDS); 10% 2-mercaptoethanol; 0.1% bromophenol blue], and 1 μg of protein per sample was separated by 12.5% SDS-polyacrylamide gel electrophoresis (PAGE) and transferred to a polyvinylidene difluoride (PVDF) membrane. The membrane was blocked with PVDF Blocking Reagent for Can Get Signal^®^ (Toyobo, Osaka, Japan) for 2 h and probed with the indicated primary antibodies to the following proteins: SOD-1 (1:1000; Enzo Life Science, Farmingdale NY, USA); SOD-2 (1:1000; Enzo Life Science); and beta-actin (1:1000; Sigma, St. Louis, MO, USA). Incubation of PVDF membranes with primary antibody was performed overnight at 4°C. After washing with Tris-Buffered Saline + Tween-20 (TBST), the membranes were further incubated with horseradish peroxidase-labeled secondary antibodies (1:1000; GE Healthcare, Amersham, UK) for 1 h at room temperature. The immobilized and labeled specific antigen was visualized with the ECL prime detection kit (GE Healthcare). Signal intensities were quantified using the Image J program and normalized to those of beta-actin.

### Cell culture

Murine cornea epithelial cells (TKE2; European Collection of Authenticated Cell Cultures, Salisbury, UK) were cultured in defined-keratinocyte serum-free medium (DKSFM; Life Technologies, Carlsbad, CA, USA) supplemented with growth supplements as provided by the manufacturer, plus 10 ng/mL EGF (Life Technologies), 0.1 μg/mL cholera toxin (List Biologic Laboratories Inc., Campbell, CA, USA), and 0.25% (w/v) penicillin–streptomycin (Life Technologies) at 37°C in a humidified atmosphere of 5% CO_2_. For subculture, cells were harvested at 80–90% confluence using TrypLE Express (Invitrogen, Carlsbad, CA, USA), subcultured at a density of 1.0 × 10^4^ cells/cm^2^, and monitored for viability by Trypan blue exclusion (Nacalai Tesque, Kyoto, Japan). The procedure was repeated for 10 passages.

### Detection of reactive oxygen species in TKE2

TKE2 were seeded in DKSFM culture medium at a density of 1.0 × 10^4^ cells/well in 96-well plate, at 37°C, in 5% CO_2_ and cultured overnight. Intracellular reactive oxygen species (ROS) levels were detected by using 5-(and-6)-chloromethyl-2′, 7′-dichlorodihydrofluorescein diacetate, acetyl ester (CM-DCFH_2_DA; Life Technologies). CM-DCFH_2_DA and quercetin were each diluted in dimethyl sulfoxide (DMSO: Wako pure chemical, Osaka, Japan) to obtain 1 mM stock solutions, which were serially diluted to make working solutions prior to application to TKE2 cells. Treatments with CM-DCFH_2_DA were for 1 h, followed by washes with PBS to remove extracellular CM-DCFH_2_DA. The cells were then stimulated by H_2_O_2_ for 1 h, before incubation with culture medium containing quercetin for 1 h. After incubation, the fluorescence (excitation 485 nm, emission 538 nm) in each well was measured using an ARVO multilabel counter (PerkinElmer; Boston, MA, USA).

### Cell viability assay

Suspended cells were seeded into a 96-well plate (2.0 × 10^3^ cells/well) and cultured overnight. The cells were stimulated by H_2_O_2_ at 37°C for 72 h, followed by quercetin treatment for 1 h. Cell viability assessed using water-soluble tetrazolium salt (WST)-8 (Dojin Chemical Co., Ltd., Kumamoto, Japan) added to each well according to the manufacturer’s instructions. After 3 h, the absorbance at 450 nm in each well was measured by absorption spectrometer (ARVO multilabel counter).

### Human study

The protocol was approved by the ethics committee of the Tokyo Dental College Ichikawa hospital, Chiba, Japan (#273). All study subjects signed written informed consent prior to enrollment. This study was registered with UMIN (UMIN000037092). Date of registration (17/06/2019).

### Onion powder test agents

Onions contain polyphenolic flavonoid compounds, especially quercetin glycosides. We used two kinds of freeze-dried onion powder, with different quercetin content. The “Quergold” onion cultivar containing a high concentration of quercetin (quercetin glycosides equivalent to 4.4 mg/g whole onion powder) was developed by the National Agriculture and Food Research Organization (NARO) (19). The “Mashiro” onion variety, with less than 0.28 mg quercetin aglycone per g, was used as the control. Volunteers ingested either of the onion powder preparations with hot soup.

### Pharmacokinetic evaluation

The pharmacokinetics associated with the two different levels of quercetin (Quergold or Mashiro) intake were also assessed by measuring the quercetin concentration in serum samples. Samples were collected from healthy volunteers, after fasting overnight, Pre (before quercetin supplementation from onion powder), 1, 2, 6, and 24 h after intake of each variety of onion powder (200 g). All samples were stored frozen until further processing.

### Crossover-design prospective trial in healthy volunteers

This was a double-blind, crossover-design prospective study of the biochemistry, functional efficacy, and tear distribution effects of intake of the two different varieties of onion powder (23 g/day) every day for 5 days. We investigated changes in quercetin concentration in tears, as well as tear function in both eyes of 7 healthy (that is, without any dry eye symptoms) volunteers (4 males, 3 females; mean age, 28.9 ± 1.3 years) by performing Schirmer 1 tear production tests, tear film breakup time (BUT) measurements, fluorescein corneal staining, and administering a questionnaire (Dry Eye related Quality of life Score: DEQS) (20) after 5 days of onion powder intake, provided in food as described above.

Two groups of randomly divided healthy volunteers were provided with onion powder with either high (Quergold) or low (Mashiro) quercetin content, ingested daily for 5 days, with food, as described above. At the end of this treatment period, and after tear samples were isolated and tests were undertaken, volunteers were subjected to a 3-day “washout” period before the onion powder test articles were switched between the two groups, and a similar 5-day administration period ensued. Finally, an additional set of samples were collected and each test described above was repeated.

### Tear collection

With the use of a micro-capillary tubes (Drummond, PA, USA) tears were collected gently from the external canthus, taking precaution to avoid reflex tearing. Tear samples were collected before the vital staining examination. Following collection, tears were placed in tubes and centrifuged at 10,000 rpm for 5 min at 4°C. The supernatants then were stored at −80°C until measured.

### Determination of the concentration of quercetin and its metabolite in serum and tears

Frozen serum or tear samples were thawed on ice. The internal standard solution (daidzein-d4) was added to each serum sample (20 μL) or tear sample (20 μL) for a final concentration of 125 pmol/mL daidzein for quercetin analyses. After the addition of ascorbic acid, samples were enzymatic hydrolyzed in phosphate buffer (pH 5.3, 500 μL) for 2 h at 37°C. After the hydrolysis, quercetin was extracted by ethyl acetate (2 × 1 mL added per sample) and samples were concentrated using a centrifugal evaporator. Each dried extract was redissolved in 20 μL of 20% acetonitrile containing 0.1% acetic acid, and 5 μL of this solution was subjected to LC-MS/MS experiments. An HPLC system (SI-2, Shiseido, Tokyo, Japan) connected to a quadruple MS/MS system API 4000 Qtrap (AB SCIEX, CA, USA) was used, and data acquisition and mass spectrometric evaluation were conducted by Analyst 1.5.1 software (AB SCIEX). HPLC gradient conditions for the quercetin were as follows: Using 1% formic acid (solution A) and acetonitrile containing 1% formic acid (solution B) the ratio of B was increased from 10 to 55% over 15 min linearly at 0.2 mL/min on a Zorbax300 SB-C8 column (2.1 mm × 150 mm, 5 um, Agilent Technologies) at 40°C. Selected reaction monitoring (SRM) was used to perform mass spectrometric quantification of quercetin (m/z 301/151 for quercetin, m/z 315/300 for isorhamnetin). The column effluent was introduced into the mass spectrometer using electrospray ionization in the negative-ion mode with declustering potential −90 V and ion spray voltage −4,400 V. The gas temperature was 450°C. Nitrogen was used as the collision gas.

### Measurement of 8-hydroxy-2′-deoxyguanosine concentrations in tear secretions

The 8-hydroxy-2′-deoxyguanosine (8-OHdG) concentrations of the collection samples were measured in tear samples collected from before and after intake of Quergold, using antibody chip methods by Healthcare Systems (Aichi, Japan).

### Statistical analysis

All statistical analyses were performed using SAS 9.4 (SAS Institute Inc., Cary, NC, USA).

#### Mice study

All summarized data were expressed as means ± SEM. Statistical significance was calculated by Bonferroni’s correction. The groups were applied with one-way ANOVA, and Bonferroni’s correction was used for multiple comparisons. A *p*-value less than 0.05 was considered statistically significant.

#### *In vitro* study

All summarized data were expressed as means ± SEM. Statistical significance was calculated by unpaired Student’s *t*-test and Mann–Whitney *U*-test. A *p*-value less than 0.05 was considered statistically significant.

#### Human study

All summarized data were expressed as means ± SD. Statistical significance was calculated by unpaired Student’s *t*-test. A *p*-value less than 0.05 was considered statistically significant.

## Results

### Effect of quercetin in the decreased tear production model mice

**FIGURE 1:**
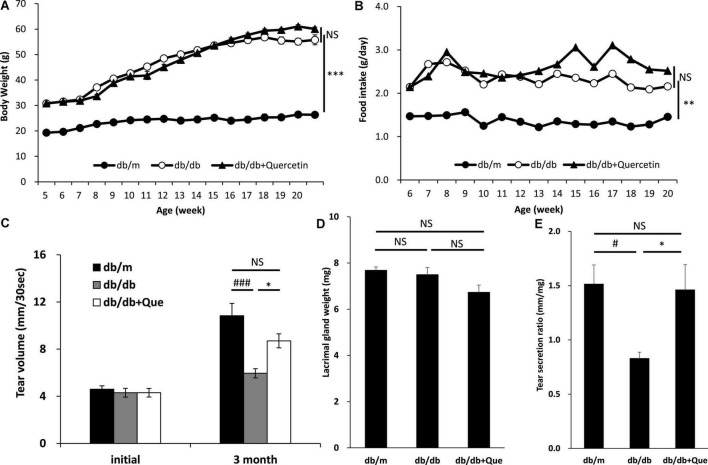
Effect of quercetin on decreased tear production in the db/db diabetic mouse model. **(A)** Time course of body weight. The mean values for body weight of db/m (*n* = 10), db/db (*n* = 10) and db/db + quercetin (*n* = 10) mice from 6 to 26 weeks of postnatal age. **(B)** Time course of food intake (g/day). Mice in the same groups as **(A)** were placed on either a CE-2 or CE-2 with 0.5% of quercetin for 26 weeks. **(C)** Basal tear volume of the db/m, the db/db, and the db/db + quercetin animals at the initiation (6 weeks of age) and 12 weeks after the start of the experiment. **(D)** Mean values for lacrimal gland weight of the db/m, the db/db, and db/db + quercetin mice. **(E)** Relative tear secretion refers to tear secretion volume adjusted for weight of each lacrimal gland in the corresponding animal. All data are presented as means ± standard error of the mean (SEM). Statistical significance was calculated by Bonferroni’s correction, ^∗∗∗^*P* < 0.001; ^∗∗^*P* < 0.01; and ^∗^*P* < 0.05 vs. db/db group, ^###^*P* < 0.001; ^#^*P* < 0.05 vs. db/m group. NS: not significant.

We examined whether treatment with quercetin improved tear secretion in leptin receptor-mutant (db/db) mice. CE-2 diet or quercetin diet was provided for 12 weeks in 6-week old female db/db mice, and food intake and body weight were serially measured over this time. The values of food intake and body weight were higher in the db/db mice than in the db/m control mice. As a result of feeding the experimental diet with 0.5% of quercetin to the db/db mice, no changes in the body weight and food intake were observed (Figures 1A,B). On the other hand, the volume of tear secretion was significantly smaller in the db/db mice than in the db/m mice (Figure 1C). The decrease in tear volume in db/db mice was significantly attenuated (56%) by treatment with quercetin (Figure 1C). No significant difference was found in the lacrimal gland weight between treatment groups (Figure 1D). Additionally, we examined the function of lacrimal gland in these mice. As shown in Figure 1E, we found that the lacrimal function, in terms of tear secretion normalized by lacrimal gland weight, was significantly lower in db/db mice than in db/m mice. However, with respect to this latter parameter, lacrimal gland function was significantly higher in quercetin treatment mice compared to db/db mice (Figure 1E).

### Histological examination of ophthalmological tissue and serum parameters

**FIGURE 2:**
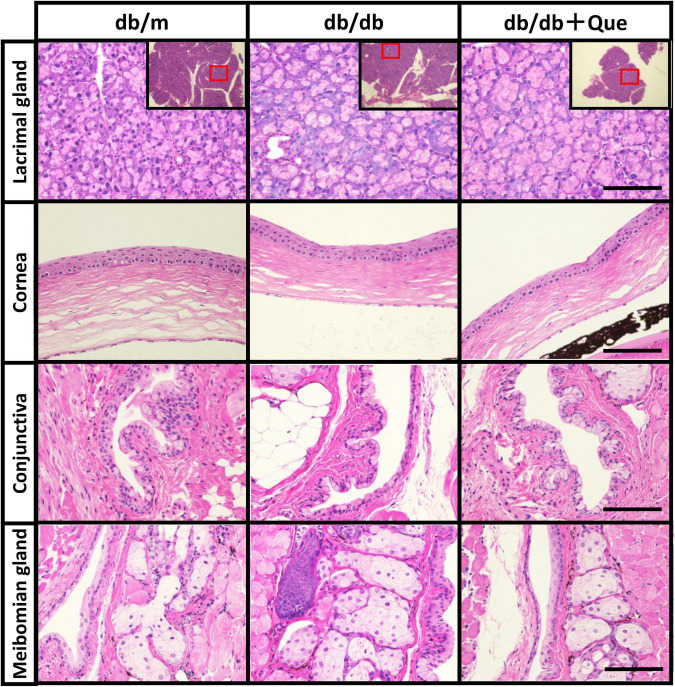
Histological examination of eye tissues. Light microscope images of hematoxylin/eosin-stained sections of the lacrimal glands, cornea, conjunctiva and meibomian glands from each experimental group were recorded. Scale bar: 50 μm.

Next, having shown that quercetin positively modulated tear production, we assessed several systemic safety effects of quercetin administration. Serum samples were collected from the all group at 12 weeks after the start of the experiment. Biochemical analysis of serum samples collected from all groups at 12 weeks after the start of the experiment showed that the levels of serum glucose and cholesterol were significantly higher in the db/db mice compared to the db/m mice. As a result of 12 weeks of feeding with the experimental diet containing 0.5% quercetin, no changes in these serum parameters were observed with db/db mice (Table 1). Further, histopathological examination of the lacrimal gland, cornea and meibomian gland showed that there were no observable changes in morphology for all groups (Figure 2). There is heavier staining in the lacrimal gland and decreased corneal thickness, for the db/db mice. Staining of lacrimal gland sections in quercetin treated db/db mice did not show any differences compare with db/db mice (size and number of acinus, density of goblet cell, thickness of cornea). These results indicated that the continuous intake of quercetin was safe.

**TABLE 1:** Biochemical serum parameters.

		db/m	db/db	*P* =	db/db + Quer	*P* =
TP	g/dL	4.6 ± 0.1	5.3 ± 0.2	^##^	5.6 ± 0.1	^###^
ALB	g/dL	3.0 ± 0.1	3.2 ± 0.1		3.5 ± 0.1	^#^
BUN	mg/dL	17.8 ± 1.6	23.6 ± 0.8		28.4 ± 2.2	^##^
CRE	mg/dL	0.1 ± 0.0	0.1 ± 0.0		0.1 ± 0.0	
Na	mEq/L	154.5 ± 0.3	152.3 ± 1.0		152.1 ± 0.6	
K	mEq/L	3.9 ± 0.1	3.6 ± 0.1		3.9 ± 0.2	
Cl	mEq/L	110.5 ± 0.9	100.9 ± 0.9	^###^	100.4 ± 1.0	^###^
Ca	mg/dL	7.5 ± 0.1	8.1 ± 0.2	^##^	8.8 ± 0.1	^###^ **
IP	mg/dL	8.0 ± 0.4	7.1 ± 0.4		7.5 ± 0.6	
AST	IU/L	78.2 ± 2.8	141.0 ± 35.6		142.4 ± 42.9	
ALT	IU/L	35.8 ± 1.9	89.6 ± 7.5		106.1 ± 23.4	^#^
LDH	IU/L	997.7 ± 125.7	1571.7 ± 272.3		1406.4 ± 200.5	
AMY	IU/L	2246.7 ± 144.2	2819.7 ± 192.1		2584.7 ± 162.7	
r-GT	IU/L	3>	3>		3>	
T-CHO	mg/dL	61.0 ± 2.7	110.0 ± 6.7	^###^	126.3 ± 4.4	^###^
F-CHO	mg/dL	14.2 ± 0.9	23.4 ± 1.4	^###^	27.0 ± 1.7	^###^
E-CHO	mg/dL	46.8 ± 2.0	86.6 ± 5.5	^###^	99.3 ± 4.8	^###^
E/T	%	76.7 ± 1.0	78.7 ± 0.7		78.6 ± 1.7	
TG	mg/dL	50.0 ± 5.7	41.7 ± 5.7		45.1 ± 5.2	
PL	mg/dL	120.8 ± 5.4	176.3 ± 8.1	^###^	209.6 ± 7.0	^###^ *
NEFA	mEq/L	1429.0 ± 125.9	1131.0 ± 51.5		1125.7 ± 70.2	
LDL-C	mg/dL	4.5 ± 0.5	8.0 ± 1.3		7.6 ± 0.6	
HDL-C	mg/dL	36.0 ± 1.6	63.7 ± 3.2	^###^	70.6 ± 3.1	^###^
T-BIL	mg/dL	0.10	0.06		0.07	^#^
TBA	mmol/L	6.5 ± 1.5	4.6 ± 1.0		4.1 ± 1.0	
TL	mg/dL	195.8 ± 11.9	298.1 ± 13.0	^###^	339.3 ± 12.8	^###^
GLU	mg/dL	24.7 ± 6.2	293.0 ± 43.5	^###^	278.4 ± 38.5	^###^
T-KB	mmol/L	3178.3 ± 252.3	872.4 ± 77.5	^###^	1043.4 ± 218.5	^###^

Serum samples were collected from the db/m, the db/db mice and db/db + Quercetin (db/db; Quer) mice (each group, *n* = 7) at 20 weeks after the start of the experiment. TP, total protein; ALB, albumin; BUN, blood urea nitrogen; CRE, creatinine; Na, sodium; K, potassium; C, chloride; Ca, calcium; IP, inorganic phosphorus; AST, aspartate aminotransferase; ALT, alanine aminotransferase; LDH, lactate dehydrogenase; AMY, amylase; r-GT, gamma-glutamyl transpeptidase; T-CHO, total cholesterol; F-CHO, free cholesterol; E-CHO, cholesteryl esters; E/T, E-CHO/T-CHO; TG, triglycerides; PL, phospholipid; NEFA, non-esterified fatty acid; LDL-C, low-density lipoprotein cholesterol; HDL-C, high-density lipoprotein cholesterol; T-BIL, total bilirubin; TBA, total bile acid; TL, total lipid; T-KB, glucose (GLU) and Total ketone body. Each value represents the mean ± SEM. Statistical significance was calculated by Bonferroni’s correction, **p < 0.01, *p < 0.05 vs. db/db group. ^###^*p* < 0.001, ^##^*p* < 0.01,^ #^*p* < 0.05 vs. db/m group.

### Induction of anti-oxidant proteins such as superoxide dismutase-1 and superoxide dismutase-2 in the lacrimal glands by quercetin treatment

**FIGURE 3:**
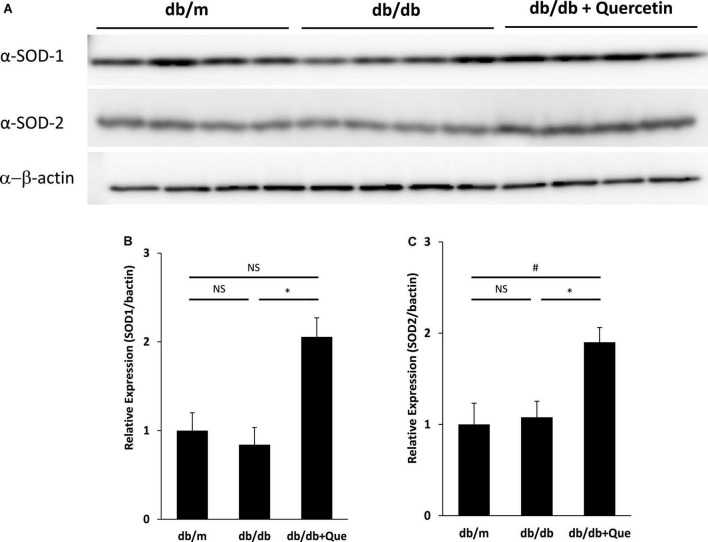
Modulation of SOD in lacrimal glands by quercetin treatment. **(A)** Western blot analysis of lacrimal glands from the db/m, the db/db, and db/db + quercetin mice (each group, *n* = 4), using antibodies to SOD-1, SOD-2 and beta-actin. Relative protein levels of SOD-1 **(B)** or SOD-2 **(C)** normalized to beta-actin in whole tissue lysates. All data are presented as means ± SEM. Statistical significance was calculated by Bonferroni’s correction, ^∗^*P* < 0.05 vs. db/db group, ^#^*P* < 0.05 vs. db/m group. NS: not significant.

Having demonstrated that quercetin treatment improved tear volume in the absence of treatment-induced pathology, we proceeded to analyze changes in expression of anti-oxidant proteins in the lacrimal gland, to correlate these observations with possible changes in quality of tear. It has been reported that SOD-1 knockout mice have oxidative stress–related damage in the lacrimal glands culminating in a decrease in tear secretion capacity. The expression analysis included the analysis of SOD-1 and SOD-2. As shown in Figure 3, we found that the expression of these anti-oxidant proteins was significantly higher in quercetin treated db/db mice than in untreated db/db mice.

### Anti-oxidant effect of quercetin in corneal epithelial cell lines exposed to oxidative stress

**FIGURE 4:**
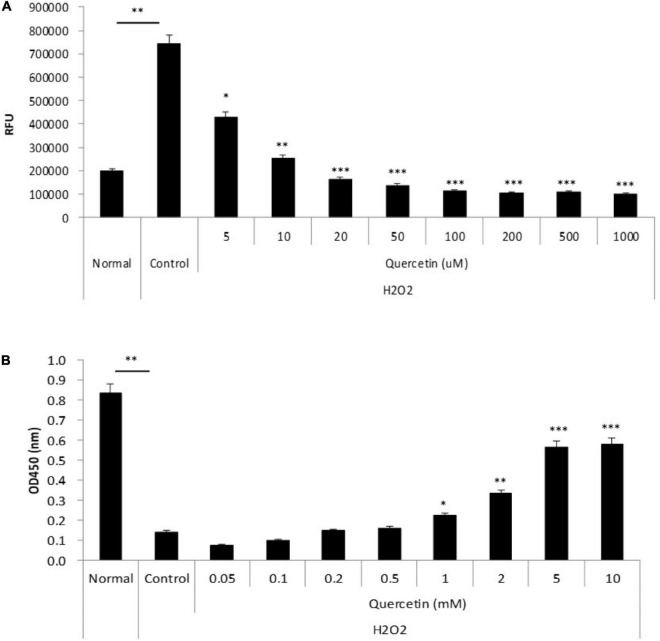
Anti-oxidant effect of quercetin by oxidative stress *in vitro*. **(A)** TKE2 cells were seeded into 96-well plates (1 × 10^4^ cells/well) and cultured for 2 days. After quercetin and H_2_O_2_ treatment, ROS production was detected by measurement of the fluorescence intensity using a plate reader. **(B)** TKE2 cells were seeded into 96-well plates (2 × 10^3^ cells/well) and cultured overnight. The cells were challenged with H_2_O_2_ at 37°C for 72 h, and then treated with quercetin solution for 1 h. Cell viability of cell was assayed using WST-8. All data are presented as means ± SEM. Statistical analysis was carried out by the Mann–Whitney *U*-test, ^∗∗∗^*P* < 0.001, ^∗∗^*P* < 0.01, and ^∗^*P* < 0.05.

To determine whether quercetin-induced expression of antioxidant proteins was associated with attenuation of ROS production, we incubated TKE2 cells, a mouse corneal epithelial cell line, with various concentrations of quercetin for 1 h, and ROS were detected by CM-DCFH_2_DA. ROS production was elevated by approximately threefold H_2_O_2_ stimulation compared with normal controls. As shown in Figure 4A, 5 μM and over of quercetin reduced ROS production to levels that were statistically significant vs. control values, and the overall response to quercetin treatment was dose-dependent. Furthermore, as shown in Figure 4B, quercetin improved cell viability of TKE2 cells challenged by the oxidative stress, as determined by cell number using the WST-8 reagent. Cell counts were reduced to 17.2% as a result of H_2_O_2_ treatment compared to normal controls. Quercetin treatment dose-dependently improved cell survival in H_2_O_2_-treated cultured (Figure 4B).

### Transition of quercetin into serum and tears following onion powder intake in healthy human volunteers

**FIGURE 5:**
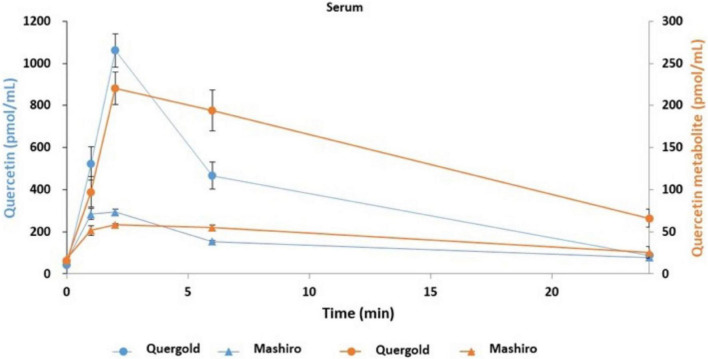
Quercetin and its metabolites concentration in serum samples from human volunteers following ingestion of high and low quercetin content onion powders. The pharmacokinetics associated with onion powder containing two different concentrations of quercetin intake were also assessed to determine the quercetin concentration in serum samples. Serum concentrations of quercetin and its metabolite in healthy volunteer intaked a single 200 mg onion sample under fasting conditions. The circles indicate the serum of subjects with high quercetin (Quergold) intake, the triangles indicate the serum of subjects with low quercetin (Mashiro) intake. The distribution of quercetin and its metabolite shifted from serum to tear following onion intake. All data are presented as means ± S.D.

Having determined that quercetin improved tear function in our mouse model, we next performed a pilot study to confirm this effect on humans. In addition, the pharmacokinetics associated with orally administered onion powder containing two different concentrations of quercetin were also assessed by determining the quercetin concentration in both serum and tear samples. The quercetin concentrations in the serum of subjects with high quercetin (Quergold) intake was four times higher than those from subjects administered the lower dose of quercetin (Mashiro), for samples collected at all-time points (0, 1, 2, 6, and 24 h after consumption) (Figure 5). This held true both for quercetin itself and its metabolite. One of the metabolites, quercetin-3-glucuronide (Q GlcA), show an antioxidant effect in human plasma. Interestingly, the concentration of quercetin metabolite in the tears were 11.9 times higher in the high quercetin content samples than in the samples representing lower quercetin content intake (Table 2).

**TABLE 2:** Transition of quercetin and its metabolite into tears following intake of onion powder by human volunteers.

	Mashiro		Quergold	
	Pre	Post	Pre	Post
Quercetin (pmol/mL)	1.23	0.99	1.41	1.24
Q GlcA (pmol/mL)	1.76	0.96	1.31	15.62

The transition into tear with orally administered 23 mg onion powder containing two different concentrations of quercetin for 5 days were also assessed by determining the quercetin concentration in tear samples. Quercetin and its metabolite in tear samples from healthy volunteers were measured by LC-MS/MS. Concentration of quercetin and its metabolite before onion powder intake in tear. Quercetin-3-Glucuronide (Q GlcA).

### Effect of quercetin on crossover-design prospective trial in healthy volunteers

**FIGURE 6:**
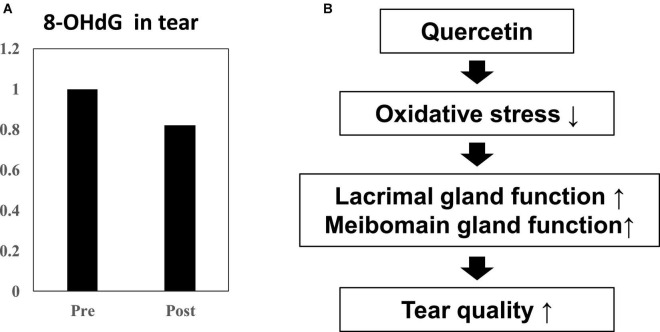
Effect of quercetin supplementation on 8-OHdG, a by-product of oxidative stress, measured in tears from healthy volunteers. **(A)** Tear samples were collected before and after intake (*n* = 4). Relative 8-OHdG levels normalized to before onion intake in tear. Ingestion of high dose quercetin in onion powder decreased the level of the oxidative stress marker 8-OHdG in tear. **(B)** Scheme of improvement in tear film stability. The distribution of quercetin and its metabolite shifted from serum to tear following onion intake. Induction of anti-oxidant proteins such as SOD-1 and SOD-2 in the lacrimal glands or meibomian gland by quercetin treatment. Quercetin reduced ROS production in the tear. Ingestion of higher quercetin doses effectively raised the quality of tears in normal humans.

Using double-blind crossover tests in both eyes of 7 healthy volunteers, we investigated the effect of onion powder containing two different concentrations of quercetin (Quergold or Mashiro) on individuals after continuous administration for 5 days. Because we used healthy subjects, there were no significant changes in tear volume, fluorescein corneal staining scores, or DEQS scores, both before and after quercetin supplementation from onion powder in all subjects. All values representing dry eye diagnostic criteria were in the normal range. However, the tear film breakup time (BUT), which is an important measure of tear quality, was lengthened after high dose quercetin administration (Table 3). Interestingly, tears from high dose quercetin samples also contained a decreased level of 8-OHdG that was trended vs. the low quercetin content tear samples (Figure 6A).

**TABLE 3:** Changes in tear function after consumption of onion powders with high and low quercetin content.

	Mashiro			Quergold		
	Pre	Post	*P* =	Pre	Post	*P* =
Tear volume by Schirmer test (mean ± SD mm)	13.1 ± 2.6	11.2 ± 2.2	0.31	14.5 ± 2.7	15.2 ± 2.7	0.72
Break up time (mean ± SD sec)	11.4 ± 2.8	11.1 ± 2.5	0.92	9.7 ± 1.6	16.6 ± 3.3	*0.03
Fluorescein score (mean ± SD points)	0.6 ± 0.2	0.6 ± 0.2	1	0.9 ± 0.2	1.2 ± 0.4	0.39
DEQS (mean ± SD points)	6.1 ± 3.4	5.0 ± 2.5	0.42	7.8 ± 5.3	2.8 ± 1.5	0.43

Healthy volunteers were tested for tear function by intake of onions with different quercetin concentrations. The measurement by performing Schirmer 1 tear production tests, tear film breakup time (BUT) measurements, fluorescein corneal staining, and administering a questionnaire (Dry Eye related Quality of life Score: DEQS) were performed before and after of onion powder intake. Each value represents the mean ± SD. *p < 0.05 vs. pre value (Student’s *t*-test).

## Conclusion

Our results showed that quercetin improved tear function through its effects on the lacrimal gland in mice and humans. Our finding that quercetin treatment induced increased expression of the anti-oxidant proteins SOD-1 and SOD-2 in the lacrimal gland suggests one possible mechanism for this improvement in tear function. In addition, quercetin inhibited ROS production and promoted cell survival in response to H_2_O_2_-induced oxidative stress in a mouse corneal epithelial cell line. Previous studies have shown that tear volume is reduced in SOD-1 knockout mice, which implicates excessive oxidative stress levels and increased ROS-mediated lacrimal gland alterations (15). Increased oxidative stress was also associated with the accumulation of secretory vesicles in acinar epithelial cells, leading to decreased tear production (15, 21, 22). Increased cellular respiration associated with ATP (Adenosine-5′-triphosphate) synthesis generates the production of free radicals by redox reactions within the mitochondrial electron transport chain (23). In the process of the respiration, ROS including superoxide (O2-), hydrogen peroxide (H_2_O_2_), hydroxyl radical (HO), singlet oxygen (^1^O_2_), lipid peroxyl radical, and hypochlorous acid (HOCl), are produced as oxygen-derived intermediates (24). ROS accelerate cellular senescence by oxidizing nucleic acids, lipids, and proteins (25–27). Prevention of cell senescence in the lacrimal gland, or even selective elimination of senescent cells damaged by oxidative stress, may be a novel therapeutic strategy to improve tear quality.

In this study, we demonstrated that following ingestion of onion powder, both quercetin as well as its metabolite were dose-dependently detected in tears and serum, and their concentrations in serum and tear were significantly higher following the high-dose quercetin onion powder intake compared to the levels detected from intake of lower doses quercetin. Interestingly, 8-OHdG in the tear sample ingested the high-dose quercetin onion powder tended to be low. Animal models with increased oxidative stress, such as SOD-1 deficient mice, have also been reported to cause abnormalities in the lacrimal glands and meibomian glands, causing dry eye (15, 28). Thus, oxidative stress in the lacrimal glands or meibomian are closely associated with dry eye, and it is possible that tear BUT improved because of Quergold improving the lacrimal gland function or meibomian gland function. Our results provided the hypothesis that the reduction of oxidative stress by quercetin improves the function of the lacrimal glands or meibomian glands, resulting in improved tear function (Figure 6B). Ingestion of higher quercetin doses effectively improved the quality of tears in humans may provide guidance in the diagnosis and prevention of dry eye. On the other hand, since the human study is a pilot study conducted by normal healthy volunteers rather than dry eye patients, the number of cases is small, there are many limitations, and it is not suitable for quantitative analysis. Although quercetin improves lacrimal gland function in mice with reduced tear secretion, it may have been difficult to confirm all these effects in normal volunteers with no change in tear volume. Further studies with quercetin-rich onions are expected, as it has been reported in humans to prevent short BUT-type dry eye by reducing the oxidative stress of the lacrimal gland function unit (29). In the future, whether quercetin improves lacrimal gland function or meibomian gland function should be examined in dry eye patients.

The anti-oxidative effect of quercetin has gained recent attention owing to its potential to prevent dry eye syndrome (30), for which important risk factors include aging and oxidative stress (31, 32). Based on the results of our research, quercetin clearly transfers into the tear compartment and can be expected to exert a preventative effect with respect to various eye disorders (30, 32, 33). These results are promising with respect to future tests on dry eye patients with reduced tear quality and volume. In addition, the concentration of quercetin that transfers to the tear after consuming foods that contain quercetin is sufficient to show local efficacy, broadening the potential diagnostic applications and suggesting a potentially useful indicator for the beneficial effects documented here, using tear samples, which can be collected non-invasively for rapid analysis (34, 35). These results may contribute to the improvement of the clinical condition of dry eye, and suggests the importance of intervention trials using optimized consumption of food sources containing various quercetin concentrations, with quercetin determination in tears as a diagnostic indicator.

Our laboratory has been studying age-related diseases by conducting epidemiological research and large clinical trials, and we have shown that administration of antioxidant vitamins and minerals significantly alleviates the symptoms of age-related eye diseases (36, 37). We have also confirmed that the anti-oxidizing properties of polyphenols and xanthophyll are effective in preventing and/or alleviating various eye diseases by their actions at the cellular level, with additional studies expected to lead to future clinical applications (6, 13, 14). Consequently, the strategy of attenuating oxidative stress by consuming nutrient-dense foods and/or dietary supplements, including quercetin, as exemplified by the results reported here, is recognized to be important to alleviate various eye diseases. Early treatment including proactive dietary intervention may be beneficial for maintaining optimal visual function.

## Data availability statement

The original contributions presented in this study are included in the article/supplementary material, further inquiries can be directed to the corresponding author.

## Ethics statement

The studies involving human participants were reviewed and approved by the Tokyo Dental College Ichikawa hospital, Chiba, Japan (#273). This study was registered with UMIN (UMIN000037092). Date of registration (17/06/2019). The patients/participants provided their written informed consent to participate in this study. The animal study was reviewed and approved by Japanese Physiological Society’s guidelines for animal care and the animal experimental committees at KEIO University (approval number: 08067).

## Author contributions

TaI, MO-K, MK, JS, and KT conceived and designed the experiments. TaI, MO-K, YL, ST, ToI, YT, and KH performed the experiments. TaI, MO-K, and ToI analyzed the data. MK contributed reagents, materials, and analysis tools. TaI and KT wrote the manuscript. All authors contributed to the article and approved the submitted version.
